# Insights about the epidemiology of *Salmonella* Typhimurium isolates from different sources in Brazil using comparative genomics

**DOI:** 10.1186/s13099-021-00423-7

**Published:** 2021-04-28

**Authors:** Amanda Ap. Seribelli, Patrick da Silva, Marcelo Ferreira da Cruz, Fernanda de Almeida, Miliane R. Frazão, Marta I. C. Medeiros, Dália dos P. Rodrigues, Jalusa D. Kich, Leandro de Jesus Benevides, Siomar de C. Soares, Marc W. Allard, Juliana Pfrimer. Falcão

**Affiliations:** 1 Departamento de Análises Clínicas, Toxicológicas e Bromatológicas, Faculdade de Ciências Farmacêuticas de Ribeirão Preto, Universidade de São Paulo - USP, Ribeirão Preto, Brazil; 2 Faculdade de Ciências Farmacêuticas de Araraquara, UNESP – Departamento de Ciências Biológicas, Rodovia Araraquara-Jaú Km 1, Araraquara, SP Brazil; 3 Instituto Adolfo Lutz de Ribeirão Preto, São Paulo, Brazil; 4 Fundação Oswaldo Cruz – FIOCRUZ, Rio de Janeiro, Brazil; 5 Empresa Brasileira de Pesquisa Agropecuária – Suínos e Aves – EMBRAPA, Concórdia, SC Brazil; 6 Universidade Federal do Triângulo Mineiro – UFTM, Uberaba, MG Brazil; 7 Food and Drug Administration-FDA, College Park, MD USA

**Keywords:** *Salmonella* Typhimurium, Phylogenetic trees, Protein orthologous clusters, Prophages, Efflux pumps

## Abstract

**Background:**

*Salmonella enterica* subsp. *enterica* serovar Typhimurium (*S*. Typhimurium) is an important zoonotic agent worldwide. The aim of this work was to compare genetically 117 *S.* Typhimurium isolated from different sources over 30 years in Brazil using different genomics strategies.

**Results:**

The majority of the 117 *S*. Typhimurium strains studied were grouped into a single cluster (≅ 90%) by the core genome multilocus sequence typing and (≅ 77%) by single copy marker genes. The phylogenetic analysis based on single nucleotide polymorphism (SNP) grouped most strains from humans into a single cluster (≅ 93%), while the strains isolated from food and swine were alocated into three clusters. The different orthologous protein clusters found for some *S*. Typhimurium isolated from humans and food are involved in metabolic and regulatory processes. For 26 isolates from swine the sequence types (ST) 19 and ST1921 were the most prevalent ones, and the ST14, ST64, ST516 and ST639 were also detected. Previous results typed the 91 *S*. Typhimurium isolates from humans and foods as ST19, ST313, ST1921, ST3343 and ST1649. The main prophages detected were: Gifsy-2 in 79 (67.5%) and Gifsy-1 in 63 (54%) strains. All of the *S*. Typhimurium isolates contained the *acrA*, *acrB*, *macA*, *macB*, *mdtK*, *emrA*, *emrB*, *emrR* and *tolC* efflux pump genes.

**Conclusions:**

The phylogenetic trees grouped the majority of the *S*. Typhimurium isolates from humans into a single cluster suggesting that there is one prevalent subtype in Brazil. Regarding strains isolated from food and swine, the SNPs’ results suggested the circulation of more than one subtype over 30 years in this country. The orthologous protein clusters analysis revealed unique genes in the strains studied mainly related to bacterial metabolism. *S*. Typhimurium strains from swine showed greater diversity of STs and prophages in comparison to strains isolated from humans and foods. The pathogenic potential of *S*. Typhimurium strains was corroborated by the presence of exclusive prophages of this serovar involved in its virulence. The high number of resistance genes related to efflux pumps is worrying and may lead to therapeutic failures when clinical treatment is needed.

## Background

Nontyphoidal *Salmonella* (NTS) strains have been an important enteric agent transmitted mainly by contaminated foods worldwide [1]. According to Kirk and collaborators [2], it was estimated that 153 million infections and 56,969 deaths occurred around the globe due to salmonellosis in 2010. Moreover, data from the Centers for Disease Control and Prevention (CDC), estimated that 1.35 million infections, 26,500 hospitalizations and 420 deaths occur in the United States every year due to *Salmonella* [3].

In Brazil, *Salmonella* has been the first or second most common foodborne pathogen isolated from outbreaks in recent years [4]. However, until now there are few published studies that have characterized the possible differences between Brazilian *Salmonella enterica* subsp. *enterica* serovar Typhimurium (*S*. Typhimurium) strains isolated from human, food and animal sources by whole genome sequencing (WGS).

*Salmonella* Typhimurium is one of the main *Salmonella* generalist serovar, which has been isolated from pork in Europe, Oceania, Asia and North America, from poultry in North America and Oceania, from beef in Africa, Latin America and Europe, and from seafood in Europe [5]. Therefore, this serovar has been transmitted from animals and humans in different parts of the world and is characterized as a zoonotic agent causing losses of million of dollars for the pork, poultry and beef producing industry [1, 6].

According to the CDC, *S.* Typhimurium can also infect domestic pets and recently was responsible for an outbreak linked to contact with small pet turtles that affected 35 people from nine states and generated 11 hospitalizations [7].

WGS has been more accessible in the last few years and is used for molecular characterization studies [8]. Furthermore, different phylogenetic strategies can be performed after sequencing, such as construction of phylogenetic trees based on the core genome multilocus sequencing typing (cgMLST), from single copy marker genes and from single nucleotide polymorphism (SNPs), besides comparison and analysis of orthologous protein clusters (OrthoVenn) and verification of the sequence type (ST) through multilocus sequence typing (MLST) [9–11]. In addition, it has been possible to characterize the different prophages that contribute to *Salmonella* pathogenicity including identification of genes known to have functions such as virulence, metabolism and signaling [12].

It is important to emphasize that the monitoring of resistant NTS strains has been of great importance due to its continued emergence worldwide [13, 14]. According to Jajere, multidrug resistant (MDR) *Salmonella* has been a serious public health problem because it may lead to treatment failure when the uses of antimicrobial drugs are necessary [14]. In the United States, it was estimated that 212,500 infections and 70 deaths occur due to drug resistant NTS every year [13].

It is known that hundreds of genes can confer resistance to antibiotics in NTS and some were previously described for the *S*. Typhimurium strains isolated from humans and different foods in Brazil including genes related to resistance to aminoglycosides, tetracyclines, sulfonamides, trimethoprim, beta lactams, fluoroquinolones, phenicol and macrolides [15]. However, antibiotic resistance is multifactorial and little is known about resistance genes related to efflux pumps, which can be an important factor that confers resistance to some antibiotics, such as fluoroquinolones, beta lactams, macrolides and aminoglycosides [15, 16].

The aim of this work was to compare genetically *S.* Typhimurium isolates from humans, food and swine in Brazil from over 30 years using different genomics strategies, such as phylogenetic trees, protein orthologous clusters analysis, MLST, prophages and resistance genes related to efflux pump.

## Results

### cgMLST

**Fig. 1:**
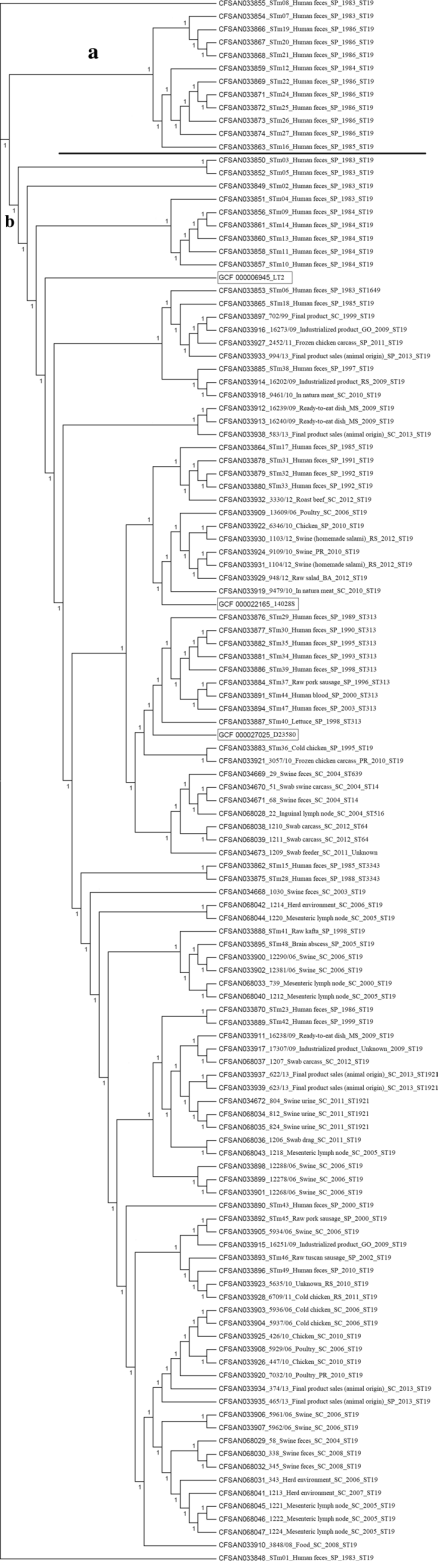
Phylogenetic analysis with cgMLST profiles based on soft core of 3002 genes selected for 117 *Salmonella* Typhimurium genomes isolated from humans (n = 43), foods (n = 48) and swine (n = 26) in Brazil

The cgMLST grouped the 120 *S.* Typhimurium genomes studied, which included the three references analysed in two main groups designated A and B (Fig. 1). Cluster A comprised 12 genomes of ST19 isolated from humans. Cluster B comprised a total of 108 genomes comprising strains isolated from humans, different foods and swine of ST19, ST1649, ST3343, ST1921 and ST313 in the case of strains isolated from humans and food, besides ST19, ST639, ST14, ST516, ST64 and ST1921 concerning strains isolated from swine. All three references were allocated in Cluster B. The CFSAN033848 and CFSAN033855 genomes isolated from humans were genetically distinct and did not group closely to any other isolates.

### Phylogenetic tree (ggTree) and orthologous protein clusters analysis

**Fig. 2:**
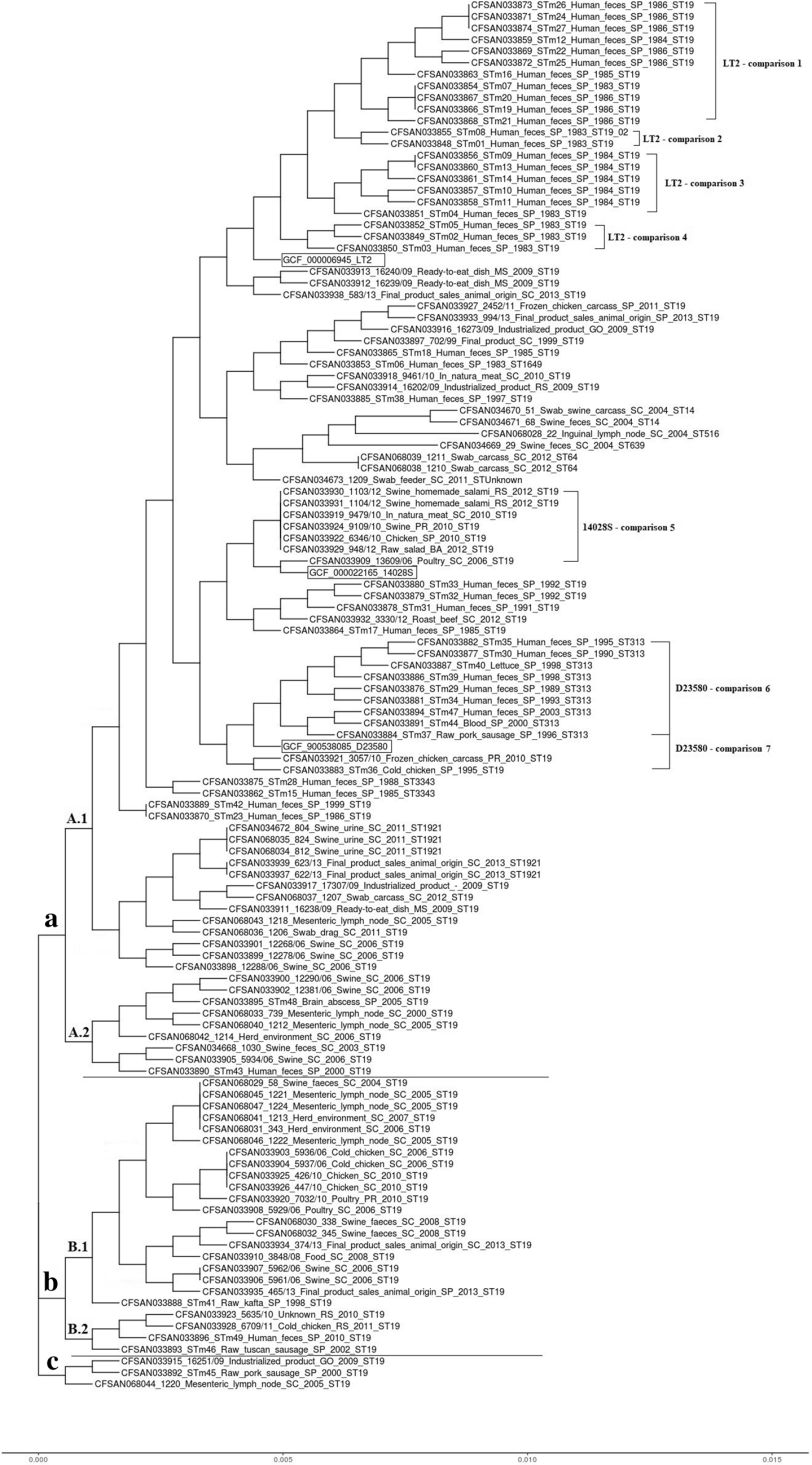
Phylogenetic analysis based on a list of single copy marker genes by ggTree for 117 *Salmonella* Typhimurium genomes isolated from humans (n = 43), foods (n = 48) and swine (n = 26) in Brazil

The ggTree grouped the 120 *S.* Typhimurium genomes studied, which included the three references analysed, in three groups designated A, B and C with cluster A subdivided in A.1 and A.2, cluster B subdivided in B.1 and B.2 (Fig. 2). Cluster A.1 comprised 84 genomes of ST19, ST1649, ST14, ST516, ST639, ST64, ST313, ST3343 and ST1921 isolated from humans, diverse foods and swine and the reference genomes. Cluster A.2 comprised nine genomes of ST19 isolated from humans, food and swine. Cluster B.1 comprised 20 genomes of ST19 from food and swine. Cluster B.2 comprised four genomes of ST19 isolated from human and food. Cluster C comprised three genomes of ST19 isolated from food and swine.

The orthologous protein clusters analysis was performed for the genomes that were more related to LT2, 14028S and D23580 references (Fig. 2). The comparisons indicated the orthologous protein clusters presented in the genomes of the strains of this study and absent in the references. The different unique orthologous protein clusters found are involved in metabolic and regulatory processes showed in detail in Table 1.

**Table 1:** Unique orthologous protein clusters in some selected *S*. Typhimurium strains in comparison to reference genomes

Groups	Biological processes (protein orthologous clusters)
LT2—STm07, STm12, STm16, STm19, STm20, STm21, STm22, STm24, STm25, STm26 and STm27 (Comparison 1)	Transposition (DNA-mediated), transposition, viral procapsid maturation, virion attachment to host cell, viral genome integration into host DNA, trehalose transport, DNA replication, viral capsid assembly, DNA binding, histidine catabolic process to glutamate and formate
LT2—STm01 and STm08 (Comparison 2)	Transposition (DNA-mediated), transposition, viral genome integration into host DNA, virion attachment to host cell, DNA replication, trehalose transport, DNA replication initiation, viral procapsid maturation, formate oxidation, DNA binding
LT2—STm04, STm09, STm10, STm11, STm13 and STm14 (Comparison 3)	Transposition (DNA-mediated), transposition, viral genome integration into host DNA, trehalose transport, histidine catabolic process to glutamate and formate
LT2—STm02, STm03 and STm05 (Comparison 4)	Transposition (DNA-mediated), transposition, viral procapsid maturation, viral genome integration into host DNA, trehalose transport, DNA replication, response to mercury ion, mercury ion transmembrane transporter activity, formate oxidation, DNA restriction-modification system
14028S—13,609/06, 6346/10, 9109/10, 9479/10, 948/12, 1103/12 and 1104/12 (Comparison 5)	Transposition (DNA-mediated), transposition, formate oxidation, trehalose transport, cell adhesion, DNA binding
D23580—STm29, STm30, STm34, STm35, STm37, STm39, STm40, STm44 and STm47 (Comparison 6)	Transposition (DNA-mediated), transposition, formate oxidation, trehalose transport, lyase activity, viral tail assembly, cell adhesion
D23580—STm29, STm30, STm34, STm35, STm36, STm37, STm39, STm40, STm44, STm47 and 3057/10 (Comparison 7)	Transposition (DNA-mediated), formate oxidation, trehalose transport, lyase activity, cell adhesion, metal ion binding

### snpTree

**Fig. 3:**
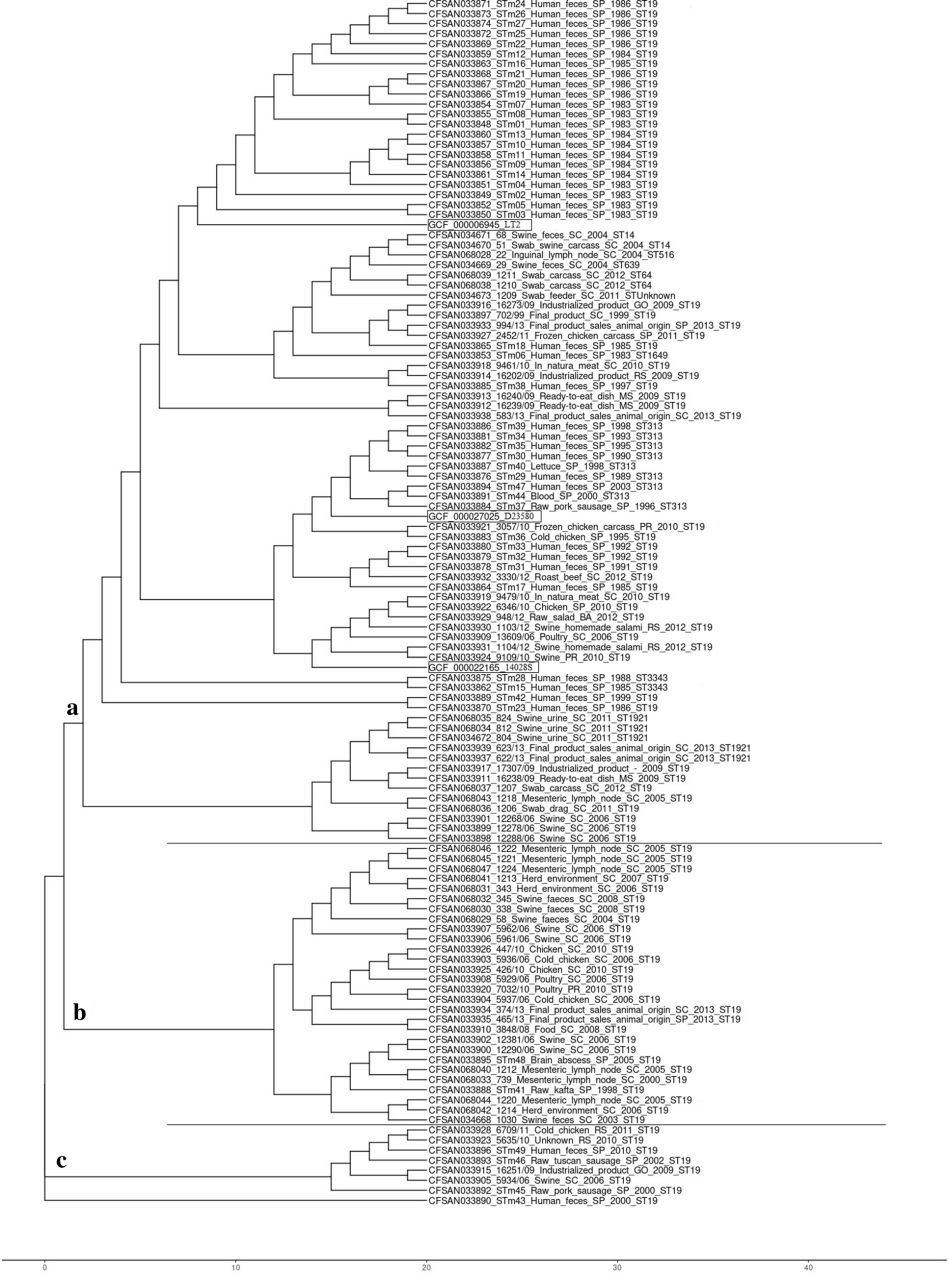
Phylogenetic analysis based on SNPs from whole genome sequencing by snpTree for 117 *Salmonella* Typhimurium genomes isolated from humans (n = 43), foods (n = 48) and swine (n = 26) in Brazil

The snpTree grouped the 120 *S.* Typhimurium genomes studied, which included the three references analysed, in three groups designated A, B and C (Fig. 3). Cluster A comprised 81 genomes of ST19, ST14, ST516, ST639, ST64, ST1649, ST313, ST3343 and ST1921 isolated from humans, food and swine, plus all three references. Cluster B comprised 28 genomes including one strain isolated from human and 27 strains isolated from different foods and swine of ST19. Cluster C comprised seven genomes including one strain isolated from human and six strains isolated from food of ST19. The CFSAN033890 genome isolated from human was genetically distinct and did not group closely to any other isolates.

### MLST

Of the 26 *S.* Typhimurium strains isolated from swine studied, 16 (61.5%) belonged to the ST19, three (11.5%) to the ST1921, two (7.6%) to the ST14, two (7.6%) to the ST64, one (3.8%) to the ST516, one (3.8%) to the ST639 and one isolate did not match any known ST type. The 91 *S*. Typhimurium isolates from humans and foods were previously typed and the ST19 (84.6%) was the most prevalent, followed by the ST313 (9.9%), ST1921 (2.2%), ST3343 (2.2%) and ST1649 (1.1%) as described in Almeida et al. [19].

### Prophages detection

The Gifsy-2 prophage was detected in 79 (67.5%) *S*. Typhimurium isolates, Gifsy-1 in 63 (54%), Salmon 118970_sal3 in 46 (39%) and Haemop—HP1 in 21 (18%). Two dozen other prophages were also detected in the genomes studied and are described in detail in Table 2.

**Table 2:** Proportion of intact prophages detected in the 117 *Salmonella* Typhimurium studied isolated in Brazil

Prophages	Humans (n = 43) (%)	Foods (n = 48) (%)	Swine (n = 26) (%)
Aeromo_phiO18P	Not detected	01/48 (2.1)	01/26 (3.8)
Burkho_BcepMu	Not detected	01/48 (2.1)	Not detected
Edward_GF_2	02/43 (4.6)	06/48 (12.5)	Not detected
Entero_186	01/43 (2.3)	01/48 (2.1)	01/26 (3.8)
Entero_BP_4795	01/43 (2.3)	Not detected	Not detected
Entero_fiAA91_ss	Not detected	Not detected	06/26 (23.1)
Entero_mEp235	Not detected	Not detected	01/26 (3.8)
Entero_N15	03/43 (7)	Not detected	Not detected
Entero_P22	03/43 (7)	Not detected	Not detected
Entero_Tyrion	01/43 (2.3)	Not detected	Not detected
Entero_UAB_Phi20	05/43 (11.6)	Not detected	Not detected
Escher_RCS47	Not detected	Not detected	02/26 (7.7)
Gifsy_1	10/43 (23)	34/48 (71)	19/26 (73)
Gifsy_2	37/43 (86)	33/48 (68.7)	09/26 (34.6)
Haemop_HP1	18/43 (41.9)	Not detected	03/26 (11.5)
Salmon_118970_sal3	25/43 (58.1)	15/48 (31.2)	06/26 (23.1)
Salmon_118970_sal4	01/43 (2.3)	Not detected	Not detected
Salmon_epsilon34	Not detected	Not detected	01/26 (3.8)
Salmon_Fels_1	Not detected	01/48 (2.1)	Not detected
Salmon_Fels_2	04/43 (9.3)	01/48 (2.1)	04/26 (15.4)
Salmon_RE_2010	07/43 (16.3)	02/48 (4.2)	01/26 (3.8)
Salmon_SEN34	Not detected	Not detected	01/26 (3.8)
Salmon_SP_004	01/43 (2.3)	04/48 (8.3)	01/26 (3.8)
Salmon_SPN1S	17/43 (39.5)	Not detected	Not detected
Salmon_SPN3UB	Not detected	Not detected	02/26 (7.7)
Salmon_SPN9CC	Not detected	03/48 (6.2)	Not detected
Salmon_SSU5	Not detected	02/48 (4.2)	01/26 (3.8)
Salmon_ST64T	01/43 (2.3)	02/48 (4.2)	Not detected
Shigel_Sfll	Not detected	01/48 (2.1)	01/26 (3.8)

### Efflux pumps

The *acrA*, *acrB*, *macA*, *macB*, *mdtK*, *emrA*, *emrB*, *emrR* and *tolC* genes were detected in the 117 (100%) *S*. Typhimurium studied. The *mdsA* and *mdsB* genes were detected in 91 (100%) *S*. Typhimurium isolates from humans and different foods, but in only 18 (69.2%) *S*. Typhimurium isolates from swine. The *mdfA* gene was detected in 26 (100%) isolates from swine, 39 (81.2%) isolates from food and 18 (42%) isolates from humans. Finally, the *cmlA1* gene was detected only in isolates from swine 05 (19.2%). The percentage of query cover and identity for all genes ranged between 72 and 100 and 87–100, respectively (Table 3).

**Table 3:** Frequencies of resistance genes related to efflux pumps in the 117 *Salmonella* Typhimurium studied

Genes	Humans (n = 43) (query cover %) (identity %)	Food (n = 48) (query cover %) (identity %)	Swine (n = 26) (query cover %) (identity %)
*acrA*	43/43 (100) (91.69)	48/48 (100) (91.69)	26/26 (100) (91.69)
*acrB*	43/43 (100) (94.66)	48/48 (100) (94.66)	26/26 (100) (94.66)
*macA*	43/43 (100) (88.65–88.84)	48/48 (100) (88.65–88.84)	26/26 (100) (88.39–88.84)
*macB*	43/43 (100) (88.60–88.70)	48/48 (100) (88.70)	26/26 (100) (88.29–88.70)
*mdtK*	43/43 (100) (99.79)	48/48 (100) (99.79)	26/26 (100) (99.16–99.79)
*emrA*	43/43 (100) (99.92–100)	48/48 (100) (100)	26/26 (72–100) (98.40–100)
*emrB*	43/43 (100) (95.7)	48/48 (100) (95.7)	26/26 (99.80–100) (95.7)
*emrR*	43/43 (100) (93.14)	48/48 (100) (93.14)	26/26 (100) (93.14)
*tolC*	43/43 (99–100) (100)	48/48 (100) (100)	26/26 (100) (98.78–100)
*mdsA*	43/43 (100) (99.75–100)	48/48 (100) (100)	18/26 (100) (100)
*mdsB*	43/43 (100) (100)	48/48 (100) (100)	18/26 (100) (100)
*mdfA*	18/43 (100) (87.93)	39/48 (100) (87.93)	26/26 (100) (87.93)
*cmlA1*	Not detected	Not detected	05/26 (100) (99.76)

## Discussion

In this study, 117 *S*. Typhimurium isolates from humans (n = 43), food (n = 48) and swine (n = 26) in Brazil were compared using genomic analyses, such as phylogenetic trees, orthologous protein clusters detection, MLST analysis, and blast identification of prophages and resistance genes related to efflux pumps.

The majority of the 117 *S*. Typhimurium strains studied were grouped into a single cluster (≅ 90%) by the core genome multilocus sequence typing and (≅ 77%) by single copy marker genes (Figs. 1 and 2). The phylogenetic analysis based on single nucleotide polymorphism (SNPs) grouped most strains from humans into a single cluster (≅ 93%), while the strains isolated from food and swine were grouped into three clusters (Fig. 3). Therefore, snpTree was more efficient at discriminating *S*. Typhimurium isolates from swine and different foods in Brazil.

It is important to mention that the present study provided additional information about *S.* Typhimurium strains isolated from humans, food and swine in Brazil because such strains have rarely been studied in a one health perspective combining all available data [15, 17–19].

Previous studies performed by our research group using different molecular typing techniques (PFGE, MLVA and CRISPR-MVLST) and a SNP-based tree by the CFSAN pipeline corroborated with the finding of snpTree by CSI Phylogeny 1.4. indicating the possible presence of a prevalent subtype for *S*. Typhimurium strains isolated from humans and with more than one circulating subtype for strains isolated from food [15, 17–19].

According to Jensen, homologous genes can be divided into orthologous and paralogs genes [20]. Orthologous genes originated from a common ancestor during speciation events and keep the same function, while, paralogs genes originated from duplication events and do not maintain the same function [21].

Therefore, the OrthoVenn2 is a web server capable to annotate and compare orthologous protein clusters from the whole genome among different species [21]. In the present study, *S*. Typhimurium genomes were compared to LT2, 14028S and D23580 references and had their unique protein orthologous clusters determined (Fig. 2). All *S*. Typhimurium isolates compared to LT2 (Comparisons 1, 2, 3 and 4) were composed of ST19 isolated from humans in the São Paulo State before the 1990s. There were some unique orthologous protein clusters, including transposition (DNA-mediated), transposition, viral genome integration into host DNA and Trehalose transport that were commonly present in these strains, but absent in the corresponding LT2 reference strain. The *S*. Typhimurium isolates compared to 14028S contained ST19 strains isolated from food in the Rio Grande do Sul, Santa Catarina and Bahia States between 2006 and 2012 (Comparison 5). The *S*. Typhimurium isolates compared to D23580 contained ST313 and ST19 strains isolated from humans and food in the São Paulo and Paraná States between 1995 and 2010 (Comparisons 6 and 7).

The different orthologous protein clusters found are involved in metabolic and regulatory processes, such as transposition, DNA replication, cell adhesion, formate oxidation, trehalose transport, lyase activity and response to mercury ion. These results showed that despite being of the same serovar there are unique orthologous protein clusters in the strains studied in comparison to the reference strains which were maintained in these *S*. Typhimurium strains during natural selection and adaptation (Table 1).

In this study, MLST was performed only for swine isolates, because the STs for humans and food isolates were previously described by Almeida et al. [22]. Of the 26 *S.* Typhimurium strains isolated from swine studied, 16 (61.5%) belonged to the ST19, three (11.5%) to the ST1921, two (7.6%) to the ST14, two (7.6%) to the ST64, one (3.8%) to the ST516, one (3.8%) to the ST639 and one did not have its ST detected.

Previous works showed that the ST19 was the most common ST found for strains of human and food origins, with ST313 being the second most prevalent and ST1921, ST3343 and ST1649 were also detected among these strains [22]. *S*. Typhimurium isolates from swine showed greater diversity in the seven housekeeping genes studied despite having a lower number of strains (n = 26) in comparison to the number of *S*. Typhimurium strains isolated from humans (n = 43) and food (n = 48). ST19 was the most commonly observed in swine, with ST1921 the second most prevalent and with ST14, ST64, ST516 and ST639 also observed.

For ST19 it has been reported 29,572 *Salmonella* isolated from human, reptile, ovine, swine, poultry, food and bovine from France, Mexico, China, Germany, Scotland, Portugal, Qatar, Korea, Ireland, United States (US), United Kingdom (UK) and Denmark according to the Enterobase (12/15/2020). The ST313 has been linked to 3049 samples isolated predominantly from humans in Kenya, Ethiopia, Zimbabwe, Malawi, Mali and Nigeria [10].

Moreover, ST1649, ST3343 and ST1921 were found for 16, 4 and 7 strains respectively, isolated from humans, livestocks, food and swine in Venezuela, Ireland, US, UK, Colombia, Ecuador, Vietnam and Brazil [10]. Finally, ST516, ST64, ST639 and ST14 were linked to 370, 3850, 237 and 2149 strains respectively, isolated from humans, poultry, food, aquatic animals, reptiles and the environment in the US, Mexico, Senegal, Germany, Portugal, Qatar, Canada, UK, India, Ghana, Thailand, Malaysia, Malta, Vietnam, Pakistan, Greece, France, Germany, China, Denmark, Scotland, Norway and South Korea [10].

It is important to emphasize that the classic MLST sequencing scheme uses only seven housekeeping genes to determine a sequence type (ST) from the nucleotides differences found in the sequences of all alleles [23]. Furthermore, cgMLST focuses on the nucleotide differences between the set of 3002 conserved genes of *Salmonella* genus [10]. It is known that the ST19 has been more prevalent in *S*. Typhimurium strains which causes predominantly gastroenteritis worldwide, suggesting that in the tree based on cgMLST there is a greater diversity in the 3002 conserved genes because *S*. Typhimurium strains isolated from humans of this ST were found in the cluster A and B (Fig. 1).

In the present study, the Gifsy-2 prophage was detected in 79 (67.5%) *S*. Typhimurium isolates, Gifsy-1 in 63 (54%), Salmon 118970_sal3 in 46 (39%) and Haemop—HP1 in 21 (18%). Specifically, Gifsy-1 prophage was detected in 10 (23%) *S*. Typhimurium strains isolated from humans, 34 (71%) strains isolated from different foods and in 19 (73%) strains isolated from swine (Table 2). Gifsy-2 prophage was detected in 37 (86%) *S*. Typhimurium strains isolated from humans, 33 (68.7%) strains isolated from foods and 9 (34.6%) strains isolated from swine (Table 2).

It is important to be mentioned that Gifsy prophages carry genes that are related to virulence of *S*. Typhimurium in the host [24, 25]. The Gifsy-1 prophage encodes three genes involved in the intracellular survival of *Salmonella* spp. in the host, denominated *gogB* (leucine-rich repeat protein), *sarA* (anti-inflammatory response activator) and p*agK2*. In the same way, the Gifsy-2 prophage encodes a superoxide dismutase (*sodC1*) that contributes to the survival of *Salmonella* spp. destroying the toxic radicals of the host macrophages [12, 26]. It is important to emphasize that Gifsy prophages have been found only in *S*. Typhimurium strains, as well as the Fels-1 and Fels-2 prophages [25]. In the present study, Fels prophages were detected in four *S*. Typhimurium strains isolated from humans, two strains isolated from food and four strains isolated from swine (Table 2).

According to Brussow et al. [27], the Fels-1 prophage encodes the *sodCIII* and *nanH* genes related to the production of superoxide dismutase and neuraminidase in *S*. Typhimurium, respectively. Furthermore, the Fels-2 prophage carries genes that are apparently related to regulation and adhesion of *S*. Typhimurium to host cells [12].

The Gifsy and Fels prophages have already been described in *S*. Typhimurium isolated in various parts of the world, such as Australia, Europe, China, among others [28–30]. It is important to emphasize that other prophages were also found in the *S*. Typhimurium strains studied including Salmon 118970_sal3 and Haemop—HP1 (Table 2). Moreover, two dozen other prophages were detected in the *S*. Typhimurium strains studied, but there is less information about them related to pathogenicity and/or virulence of this serovar (Table 2).

In addition, *S*. Typhimurium isolates from swine showed 6 (23.1%) unique prophages despite having a lower number of strains analysed (n = 26) in comparison to *S*. Typhimurium strains isolated humans (n = 43) and food (n = 48) that presented 7 (16.3%) and 3 (6.25%) unique prophages, respectively, suggesting the greater diversity in these mobile genetic element for *S*. Typhimurium strains isolated from swine in Brazil (Table 2).

Resistance to multiple drugs in bacteria has been a serious public health problem worldwide [31]. It is known that there are four main mechanisms that can cause this resistance, such as target alteration, drug inactivation, decreased permeability and drug expulsion through the production of efflux pumps [32].

In the present study, the *acrA*, *acrB*, *macA*, *macB*, *mdtK*, *emrA*, *emrB*, *emrR*, *tolC*, *mdsA*, *mdsB*, *mdfA* and *cmlA1* genes were detected among the *S*. Typhimurium strains isolated from humans, food and swine. All of the isolates contained the *acrA*, *acrB*, *macA*, *macB*, *mdtK*, *emrA*, *emrB*, *emrR* and *tolC* genes (Table 3). Other genes related to production efflux pump, such as *oqxAB* and *floR* were previously reported in [15].

The AcrAB efflux system has been described as responsible for the intrinsic resistance to many antibiotics that can be used in medical practice for the treatment of *S*. Typhimurium, such as fluoroquinolones and beta-lactams [16]. According to the World Health Organization (WHO), *Salmonella* spp. was described as a high priority category pathogen in fluoroquinolones resistance of the Global Priority Pathogens List [31].

The *macA* and *macB* genes encode proteins that characterize an efflux pump related to macrolides resistance [33, 34]. According to the Universal Protein Resource (UniProt), the *mdtK*, *emrA*, *emrB*, *emrR*, *mdsA*, *mdsB*, *mdfA* and *cmlA1* genes encode mainly proteins involved in multidrug efflux transporter and confers resistance to different antibiotics, such as aminoglycosides, tetracyclines, novobiocin, nalidixic acid, chloramphenicol and norfloxacin [34, 35]. Furthermore, the *tolC* gene has been described as important for the formation of some multidrug efflux systems (AcrAB, MacAB, EmrAB and MdsAB) in *S*. Typhimurium [35].

## Conclusions

The phylogenetic trees grouped the majority of the *S*. Typhimurium isolates from humans into a single cluster suggesting that there is one prevalent subtype in Brazil. Regarding strains isolated from food and swine, the results by SNPs analysis suggested the circulation of more than one subtype over 30 years in this country. The orthologous protein clusters analysis revealed unique genes in the strains studied mainly related to bacterial metabolism. *S*. Typhimurium isolates from swine showed greater diversity of STs and prophages in comparison to *S*. Typhimurium strains isolated from humans and food. The pathogenic potential of *S*. Typhimurium strains was corroborated by the presence of exclusive prophages of this serovar involved in their virulence. The high number of resistance genes related to efflux pump is worrying and may cause therapeutic failures when clinical treatment is needed. Altogether, this study provided relevant data on the genomic characterization of *S*. Typhimurium strains isolated from different sources in Brazil using WGS.

## Methods

### Bacterial strains

A total of 117 *S.* Typhimurium strains isolated from humans (43), food (48) and swine (26) between 1983 and 2013 in Brazil were studied (Table 4). These strains were selected from the collections of the Adolfo Lutz Institute of Ribeirão Preto (IAL-RP), of the Oswaldo Cruz Foundation from Rio de Janeiro (FIOCRUZ-RJ) and of the Brazilian Agricultural Research Corporation (EMBRAPA).

**Table 4:** Characteristics of the 117 *Salmonella* Typhimurium strains studied isolated from different sources in Brazil

CFSAN nº	Isolate name	Source	State	Year of isolation	Sequence type (ST)
CFSAN033848	STm01	Human feces	SP	1983	19
CFSAN033849	STm02	Human feces	SP	1983	19
CFSAN033850	STm03	Human feces	SP	1983	19
CFSAN033851	STm04	Human feces	SP	1983	19
CFSAN033852	STm05	Human feces	SP	1983	19
CFSAN033853	STm06	Human feces	SP	1983	1649
CFSAN033854	STm07	Human feces	SP	1983	19
CFSAN033855	STm08	Human feces	SP	1983	19
CFSAN033856	STm09	Human feces	SP	1984	19
CFSAN033857	STm10	Human feces	SP	1984	19
CFSAN033858	STm11	Human feces	SP	1984	19
CFSAN033859	STm12	Human feces	SP	1984	19
CFSAN033860	STm13	Human feces	SP	1984	19
CFSAN033861	STm14	Human feces	SP	1984	19
CFSAN033862	STm15	Human feces	SP	1985	3343
CFSAN033863	STm16	Human feces	SP	1985	19
CFSAN033864	STm17	Human feces	SP	1985	19
CFSAN033865	STm18	Human feces	SP	1985	19
CFSAN033866	STm19	Human feces	SP	1986	19
CFSAN033867	STm20	Human feces	SP	1986	19
CFSAN033868	STm21	Human feces	SP	1986	19
CFSAN033869	STm22	Human feces	SP	1986	19
CFSAN033870	STm23	Human feces	SP	1986	19
CFSAN033871	STm24	Human feces	SP	1986	19
CFSAN033872	STm25	Human feces	SP	1986	19
CFSAN033873	STm26	Human feces	SP	1986	19
CFSAN033874	STm27	Human feces	SP	1986	19
CFSAN033875	STm28	Human feces	SP	1988	3343
CFSAN033876	STm29	Human feces	SP	1989	313
CFSAN033877	STm30	Human feces	SP	1990	313
CFSAN033878	STm31	Human feces	SP	1991	19
CFSAN033879	STm32	Human feces	SP	1992	19
CFSAN033880	STm33	Human feces	SP	1992	19
CFSAN033881	STm34	Human feces	SP	1993	313
CFSAN033882	STm35	Human feces	SP	1995	313
CFSAN033883	STm36	Cold chicken	SP	1995	19
CFSAN033884	STm37	Raw pork sausage	SP	1996	313
CFSAN033885	STm38	Human feces	SP	1997	19
CFSAN033886	STm39	Human feces	SP	1998	313
CFSAN033887	STm40	Lettuce	SP	1998	313
CFSAN033888	STm41	Raw kafta	SP	1998	19
CFSAN033889	STm42	Human feces	SP	1999	19
CFSAN033890	STm43	Human feces	SP	2000	19
CFSAN033891	STm44	Blood	SP	2000	313
CFSAN033892	STm45	Raw pork sausage	SP	2000	19
CFSAN033893	STm46	Raw tuscan sausage	SP	2002	19
CFSAN033894	STm47	Human feces	SP	2003	313
CFSAN033895	STm48	Brain abscess	SP	2005	19
CFSAN033896	STm49	Human feces	SP	2010	19
CFSAN033897	702/99	Final product	SC	1999	19
CFSAN033898	12,288/06	Swine	SC	2006	19
CFSAN033899	12,278/06	Swine	SC	2006	19
CFSAN033900	12,290/06	Swine	SC	2006	19
CFSAN033901	12,268/06	Swine	SC	2006	19
CFSAN033902	12,381/06	Swine	SC	2006	19
CFSAN033903	5936/06	Cold chicken	SC	2006	19
CFSAN033904	5937/06	Cold chicken	SC	2006	19
CFSAN033905	5934/06	Swine	SC	2006	19
CFSAN033906	5961/06	Swine	SC	2006	19
CFSAN033907	5962/06	Swine	SC	2006	19
CFSAN033908	5929/06	Poultry	SC	2006	19
CFSAN033909	13,609/06	Poultry	SC	2006	19
CFSAN033910	3848/08	Food	SC	2008	19
CFSAN033911	16,238/09	Ready-to-eat dish	MS	2009	19
CFSAN033912	16,239/09	Ready-to-eat dish	MS	2009	19
CFSAN033913	16,240/09	Ready-to-eat dish	MS	2009	19
CFSAN033914	16,202/09	Industrialized product	RS	2009	19
CFSAN033915	16,251/09	Industrialized product	GO	2009	19
CFSAN033916	16,273/09	Industrialized product	GO	2009	19
CFSAN033917	17,307/09	Industrialized product	–	2009	19
CFSAN033918	9461/10	In natura meat	SC	2010	19
CFSAN033919	9479/10	In natura meat	SC	2010	19
CFSAN033920	7032/10	Poultry	PR	2010	19
CFSAN033921	3057/10	Frozen chicken carcass	PR	2010	19
CFSAN033922	6346/10	Chicken	SP	2010	19
CFSAN033923	5635/10	Unknown	RS	2010	19
CFSAN033924	9109/10	Swine	PR	2010	19
CFSAN033925	426/10	Chicken	SC	2010	19
CFSAN033926	447/10	Chicken	SC	2010	19
CFSAN033927	2452/11	Frozen chicken carcass	SP	2011	19
CFSAN033928	6709/11	Cold chicken	RS	2011	19
CFSAN033929	948/12	Raw salad	BA	2012	19
CFSAN033930	1103/12	Swine (homemade salami)	RS	2012	19
CFSAN033931	1104/12	Swine (homemade salami)	RS	2012	19
CFSAN033932	3330/12	Roast beef	SC	2012	19
CFSAN033933	994/13	Final product sales (animal origin)	SP	2013	19
CFSAN033934	374/13	Final product sales (animal origin)	SC	2013	19
CFSAN033935	465/13	Final product sales (animal origin)	SP	2013	19
CFSAN033937	622/13	Final product sales (animal origin)	SC	2013	1921
CFSAN033938	583/13	Final product sales (animal origin)	SC	2013	19
CFSAN033939	623/13	Final product sales (animal origin)	SC	2013	1921
CFSAN068033	739	Mesenteric lymph node	SC	2000	19
CFSAN034668	1030	Swine feces	SC	2003	19
CFSAN068028	22	Inguinal lymph node	SC	2004	516
CFSAN034669	29	Swine feces	SC	2004	639
CFSAN034670	51	Swab swine carcass	SC	2004	14
CFSAN034671	68	Swine feces	SC	2004	14
CFSAN068029	58	Swine faeces	SC	2004	19
CFSAN068040	1212	Mesenteric lymph node	SC	2005	19
CFSAN068043	1218	Mesenteric lymph node	SC	2005	19
CFSAN068044	1220	Mesenteric lymph node	SC	2005	19
CFSAN068045	1221	Mesenteric lymph node	SC	2005	19
CFSAN068046	1222	Mesenteric lymph node	SC	2005	19
CFSAN068047	1224	Mesenteric lymph node	SC	2005	19
CFSAN068031	343	Herd environment	SC	2006	19
CFSAN068042	1214	Herd environment	SC	2006	19
CFSAN068041	1213	Herd environment	SC	2007	19
CFSAN068030	338	Swine faeces	SC	2008	19
CFSAN068032	345	Swine faeces	SC	2008	19
CFSAN068034	812	Swine urine	SC	2011	1921
CFSAN068035	824	Swine urine	SC	2011	1921
CFSAN068036	1206	Swab drag	SC	2011	19
CFSAN034672	804	Swine urine	SC	2011	1921
CFSAN034673	1209	Swab feeder	SC	2011	Unknown
CFSAN068037	1207	Swab carcass	SC	2012	19
CFSAN068038	1210	Swab carcass	SC	2012	64
CFSAN068039	1211	Swab carcass	SC	2012	64

*SP *São Paulo, *SC *Santa Catarina, *MS *Mato Grosso, *RS *Rio Grande do Sul, *GO *Goiás, *PR *Paraná, *BA *Bahia

### Whole genome sequencing

The DNA of the 117 *S.* Typhimurium strains was extracted according to Campioni and Falcão using phenol-chloroform-isoamyl alcohol method [36]. Libraries were prepared using 1 ng of genomic DNA with the Nextera XT DNA library preparation kit (Illumina, San Diego, CA, USA) and the genomes were sequenced using the NextSeq 500 desktop sequencer with the NextSeq 500/500 high-output version 2 kit (Illumina) for 2 × 151 cycles according to the manufacturer’s instructions at the U.S. Food and Drug Administration (FDA), College Park, Maryland, USA. The genomes were assembled using the software SPAdes and CLC Genomics Workbench version 10.0.1 [37] and the quality of the assemblies were evaluated using the software QUAST [38]. The genomes ranged from 4.6 to 5.1 Mb in size, as described for other *Salmonella* strains [39]. Sequencing generated an average G+C content of 52.04%, which is similar to that reported previously for other *Salmonella* isolates [40]. The number of contigs per assembly for each isolate ranged between 47 and 827. Finally, the coverage (×) ranged from 13× to 753×. Detailed information on the sequencing of the 117 *S*. Typhimurium genomes can be found in Almeida et al. and Seribelli et al. [41, 42].

### cgMLST

The cgMLSTFinder 1.1 analysis was determined from a set of reads for all 117 *S*. Typhimurium genomes and three different references of this serovar were chosen, which included LT2, 14028S and D23580 and compared using the services of the center for genomic epidemiology for *Salmonella* (Enterobase) available at https://cge.cbs.dtu.dk/services/cgMLSTFinder/ [10].

### Phylogenetic tree (ggTree) and orthologous protein clusters analysis

Three different references of *S*. Typhimurium serovar were chosen, which included LT2 (GCF_000006945), 14028S (GCF_000022165) and D23580 (GCF_900538085), all with fully closed deposited genomes. To evaluate the evolutionary distance between the sequenced genomes and the three reference strains, a neighbor-joining tree was built with the ezTree algorithm [11] and ggTree R package [43, 44] (Fig. 2). The ezTree has been described as an automated pipeline based in the single copy marker genes identification to construct a phylogenetic tree for a set of input genomes [11]. Additional characterization of the orthologous protein clusters for some of the key *S*. Typhimurium strains were performed. The phylogroups selected included: Comparison 1—LT2 with 11 genomes (CFSAN033873, CFSAN033871, CFSAN033874, CFSAN033859, CFSAN033869, CFSAN033872, CFSAN033863, CFSAN033854, CFSAN033867, CFSAN033866 and CFSAN033868); Comparison 2—LT2 with two genomes (CFSAN033855 and CFSAN033848); Comparison 3—LT2 with six genomes (CFSAN033856, CFSAN033860, CFSAN033861, CFSAN033857, CFSAN033858 and CFSAN033851); Comparison 4—LT2 with three genomes (CFSAN033852, CFSAN033849 and CFSAN033850); Comparison 5—14028S with seven genomes (CFSAN033930, CFSAN033931, CFSAN033919, CFSAN033924, CFSAN033922, CFSAN033929 and CFSAN033909); Comparison 6—D23580 with nine genomes (CFSAN033882, CFSAN033877, CFSAN033887, CFSAN033886, CFSAN033876, CFSAN033881, CFSAN033894, CFSAN033891 and CFSAN033884); Comparison 7—D23580 with 11 genomes (CFSAN033882, CFSAN033877, CFSAN033887, CFSAN033886, CFSAN033876, CFSAN033881, CFSAN033894, CFSAN033891, CFSAN033884, CFSAN033921 and CFSAN033883) via OrthoVenn2 [21] in order to determine unique features and metabolic pathways defining each group.

### SNP tree

The phylogenetic tree based on SNPs of the whole genome sequencing was performed by CSI Phylogeny 1.4 (Call SNPs & Infer Phylogeny) of the Center for Genomic Epidemiology at https://cge.cbs.dtu.dk/services/CSIPhylogeny/—following the parameters: select min. depth at SNP positions 10×, select min. relative depth at SNP positions 10%, select minimum distance between SNPs (prune) 10 bp, select min. SNP quality 30, select min. read mapping quality 25 and select min. Z-score 1.96 [45]. The SNPs matrix included was a maximum of 30,873 SNPs among all *S*. Typhimurium strains studied.

### Multilocus sequence typing (MLST)

MLST was performed in the present study for the 26 *S*. Typhimurium isolates from swine using the MLST 2.0 of the Center for Genomic Epidemiology for *Salmonella enterica* available in https://cge.cbs.dtu.dk/services/MLST/ [46]. The seven housekeeping genes included: *aroC*, *dnaN*, *hemD*, *hisD*, *purE*, *sucA* and *thrA* [23, 46]. The STs of the *S*. Typhimurium isolates from humans and different foods were previously described in Almeida et al. [22] and were performed in the same way as described above.

### Prophages detection

The genomes of all 117 *S*. Typhimurium strains were used to search the prophages by PHAge Search Tool Enhanced Release (PHASTER) that is an online platform for the rapid identification and annotation of prophages sequences in bacterial genomes and plasmids available in http://phaster.ca/ [47].

### Efflux pumps

The genomes of all 117 *S*. Typhimurium strains were used to search for resistance genes related to efflux pump. Resistance gene identifier (RGI) is part of the Comprehensive Antibiotic Resistance Database (CARD) and was performed with high quality/coverage (includes contigs > 20,000 bp and excludes prediction of partial genes). Software is available at https://card.mcmaster.ca/analyze/rgi [48].

## Data Availability

The data from 117 *S*. Typhimurium genomes were deposited in the GenBank (NCBI) under the identification numbers: LVHC00000000, LVHB00000000, LVHA00000000, LVGZ00000000, LVGY00000000, LVGX00000000, LVGW00000000, MABI00000000, LVGV00000000, LVGU00000000, LVGT00000000, LUJG00000000, LVGS00000000, LVGR00000000, LVGQ00000000, LVGP00000000, LVGO00000000, LVGN00000000, LVGM00000000, LVGL00000000, LUJF00000000, LVGK00000000, LVGJ00000000, LVGI00000000, LVGH00000000, LVGG00000000, LVGF00000000, LUJE00000000, LVGE00000000, LVGD00000000, LUJD00000000, LVGC00000000, LVGB00000000, LVGA00000000, LVFZ00000000, LVFY00000000, LVFX00000000, LUJC00000000, LUJB00000000, LUJA00000000, LVFW00000000, LUIZ00000000, LVFV00000000, LVFU00000000, LUIY00000000, LVFT00000000, LUIX00000000, LUIW00000000, LVFS00000000, LVFR00000000, LUIV00000000, LUIU00000000, LUIT00000000, LVFQ00000000, LUIS00000000, LUIR00000000, LUIQ00000000, LUIP00000000, LUIO00000000, LVFP00000000, LUIN00000000, LUIM00000000, LUIL00000000, LUIK00000000, LVFO00000000, LUIJ00000000, LUII00000000, LUIH00000000, LVFN00000000, LUIG00000000, LUIF00000000, LUIE00000000, LVFM00000000, LUID00000000, LUIC00000000, LVFL00000000, LVFK00000000, LUIB00000000, LUIA00000000, LUHZ00000000, LVFJ00000000, LUHY00000000, LUHX00000000, LVFI00000000, LUHW00000000, LUHV00000000, LUHU00000000, LUHT00000000, LUHS00000000, LUHR00000000, LVFH00000000, SRR8291813, SRR8291805, SRR8291802, SRR8291817, SRR8291806, SRR8291814, PHJE00000000, PHJD00000000, PHJC00000000, PIJC00000000, PHJB00000000, PHJA00000000, PHIZ00000000, PHIY00000000, PHIX00000000, PHIW00000000, PHIV00000000, PHIU00000000, PHIT00000000, PHIS00000000, PHIR00000000, PHIQ00000000, PHIP00000000, PHIO00000000, PHIN00000000, PHIM00000000, released under the project PRJNA186035 (https://www.ncbi.nlm.nih.gov/bioproject/186035).

## Abbreviations

NTS: Nontyphoidal *Salmonella*
CDC: Centers for Disease Control and Prevention
WGS: Whole genome sequencing
cgMLST: Core genome multilocus sequence typing
SNP: Single nucleotide polymorphism
ST: Sequence type
MLST: Multilocus sequence typing
MDR: Multidrug resistant
DNA: Deoxyribonucleic acid
WHO: World Health Organization
UniProt: Universal Protein Resource
FDA: Food and Drug Administration
PHASTER: PHAge Search Tool Enhanced Release
RGI: Resistance gene identifier
CARD: Comprehensive Antibiotic Resistance Database
IAL-RP: Adolfo Lutz Institute of Ribeirão Preto
FIOCRUZ-RJ: Oswaldo Cruz Foundation from Rio de Janeiro
EMBRAPA: Brazilian Agricultural Research Corporation
